# The Progress of Dentistry

**Published:** 1879-11

**Authors:** Richard Grady


AMERICAN JOURNAL

OF

DENTAL SCIENCE.

Vol. XIII. THIRD SERIES—NOVEMBER, 1879. No. 7.

### ARTICLE I.

### *The Progress of Dentistry.*

BY RICHARD GRADY, D. D. S.

It is comparatively of late years that dentistry has occupied any thing like a properly recognized position among the different departments of the healing art. In Rees' Cyclopedia, published early in the present century, we are told that the "head surgeons in London deem this branch of their art beneath notice, and generally decline interfering in it except by giving their advice occasionally: though manual operations on the teeth and the mechanical formation of these organs in cases of defect constitute a very profitable business in such a large metropolis; so that dentists are often known to get several thousand pounds per annum by their profession!!"

For a long time dentistry was practiced to a large extent as a superadded means of livelihood by persons engaged in other pursuits, and without any professional education whatever; and it is only within a century that it has taken the rank of a distinct profession. The blacksmith, the barber, the watchmaker, the shoemaker, were the dentists of every village and country town. In 1803, the practice of making teeth and cleaning them appears to have been in the hands of silversmiths and jewelers. The explanation seems to be that mere tooth-drawing constituted the surgical dentistry of these days; and as the operation was considered one demanding muscular strength and manual dexterity more than anatomical knowledge and surgical skill, it had not many attractions for medical men, and was accordingly consigned to the uneducated and the charlatan.

Little knowledge appears to have existed in very early times concerning the causes of toothache, if we judge from a remark made by a physician in the first century, that "the cause of toothache was known only to God." However, it is claimed that the ancient Egyptians understood processes of the art of dentistry which are regarded as inventions of modern times, such as fastening artificial teeth on gold plates and filling teeth with gold and cement. But it is certain on the authority of Hippocrates (B. C. 450) that little was known concerning the anatomy, physiology and pathology of the teeth.

Galen, who flourished in the second century and next to Hippocrates, the most eminent of the ancient physicians, was the first to treat this subject; and his works were the best for a space of fourteen hundred years, or until the works of Fallopius, Eustachius and Pare were published.

From this time the treatment of diseases of the teeth received much attention. Indeed such attention was given to it that it was becoming a specialty; and prominent medical men in France and England published elaborate works devoted exclusively to dentistry. But as the writers were not practical dentists, the subject was treated theoretically. From the publication of the writings of these men, and prominently among them the treatises of Hunter, Bichat and Fauchard begins the most important epoch in the history of the rise and progress of dental science. The writings of Hunter, who was acknowledged the first practical surgeon in Christendom, laid the foundation of the English School of Dentistry, and those of Bichat, the eminent French surgeon, that of the French School of Dentistry. The progress from this time was very rapid, works being published on the principles and practice of the art, and one on Mechanical Dentistry.

Dentistry is supposed to have been introduced into the United States by Le Mair, who came from France during the Revolutionary war; but it is stated on good authority that the first dentist of whom we have any account was Wooffendale, who came from England to New York in 1766. John Greenwood, who carved two sets of teeth for Gen. Washington, which were secured by spiral springs, is believed to have been the first "American" dentist.

In the first quarter of the present century there were, perhaps, 100 dental practitioners in the United States, some few with previous education, (as Spence, Gardette, Hayden and Koecker;) but others entirely deficient in theoretical or practical knowledge; and dental surgery had made but little progress. The dentists now in the United States number 10,500, while those in other countries combined do not exceed 4,100.

With this increase in the number of dental practitioners, the progress of dentistry as a science has been very marked and rapid; and many very valuable works of a practical nature have been published, which are but the signals and registers of its onward march. In fact, no science or art, except Chemistry, has been so eminently progressive.

But this growth and development have required time, thought and talent, as well as great resources and most skilled appliances. From the simple and comparatively not-difficult operations of cleansing, extracting and filling small and superficial cavities, it has extended to a thorough and scientific treatment of the mouth, with a view not only of saving teeth but slightly decayed, but all teeth, and also anticipating decay by such operations as shall make it possible for the patient to keep the mouth thoroughly cleansed and the teeth free from the deleterious effects of fermentation of portions of food or other substances in the mouth.

It is no longer a question that dentistry is extremely useful and has done valuable service, since it is not too much to say that it adds not only to health, comfort and the enjoyment of life but in all probability many lives have been saved and a still greater number prolonged through the instrumentality of the aid afforded by the use of artificial teeth.

As with Hunter and Bichat began an important epoch in the progress of dental science in England and France, so the establishment of the American Journal of Dental Science in 1839, the formation of the Society of Dental Surgery and the founding of the Baltimore College of Dental Surgery shortly after, gave by their combined influence an impetus to dental science which it never before had, and contributed in an eminent degree, to the dignity and respectability of the profession.

There are perhaps now in existence not less than 230 National, State and Local Societies; 14 Colleges of Dental Surgery, established in the leading cities of the United States; and 8 journals devoted entirely to the interests of dentistry. And it is a matter of just pride and of memorable interest that America has the honor of nursing dentistry through its infant struggles, and that Baltimore is its birthplace, for in Baltimore was established the first institution that gave it reputation.

The mechanical branch of the science has conferred upon man the marvelous power of imitating in his laboratory the production of living nature. Formerly artificial teeth were carved from ivory. They were also obtained by altering the shape of teeth of inferior animals, and the crowns of human teeth were often conveniently engrafted on the roots of original front teeth. All these materials were objectionable from their susceptibility to the action of the fluids of the mouth. Porcelain teeth (known as "incorruptible") perfectly resist this action. They are of French origin; but their present perfection is due to American enterprise. One establishment in Philadelphia, which has spared neither labor nor expense in securing, improving and perfecting artificial teeth by employing the most skillful talent, turns out nearly 4,000,000 teeth annually; about one-half of what are made in Europe and America.

Various methods of securing artificial teeth in their places have been in use. In ancient times, they were fastened by ligatures of flax or silk, and with wire of gold or silver to the natural teeth that remained. In modern times, metallic clasps, spiral springs and fastenings of gutta percha and caoutchouc have been used for this purpose. Another method is to secure the teeth, either in whole or partial sets, to a plate of gold or other metal, which is so accurately fitted to the gums that it is retained by atmospheric pressure.

In 1851, the process called Continuous Gum was patented by Dr. John Allen, but priority of invention was contested by Dr. Wm. H. Hunter. In this, a silicious compound, similar to that of which the teeth are made, but more fusible, is applied in the form of paste over the fastenings at the back of the teeth, and also to the front, so as to bury entirely the ends of the teeth, as the natural ones are buried in the gums. To withstand the high degree of heat requisite for baking this upon the plate, platinum is substituted for gold. Platinum has besides the advantage of forming at a high heat a close union with the silicious compound. The compositions used are empirical mixtures of pure silica and feldspar, with a suitable flux to produce a fusible compound possessing sufficient strength, hardness and permanency of character. This work can be easily repaired when broken, or alterations made when required by changes in the mouth.

For 100 years, physicians sought for agents by the use of which pain in surgical operations could be avoided; and chemical science at length furnished the inestimable boon through an American dentist. As the suggestion was first made by an American dentist, so the first use of the suggestion was in dental operations. As is well known, the priority of the claim to the introduction of ether as an anæsthetic agent has been hotly contested between Dr. Wells, of Hartford, and Dr. Morton, of Boston. The anæsthetic effect of nitrous oxide was first suggested by Sir Humphrey Davy in 1776, and practically demonstrated by Dr. Horace Wells. Several years since Dr. Richardson introduced bi-chloride of methyline. Galvanism was first applied for dental anæsthesia in 1858 by Mr. Francis, a dentist of Philadelphia. Several instruments have also been invented for the application of freezing mixtures to the teeth preparatory to extraction; so that when we compare the present mode of extracting teeth with that formerly in vogue (even as late as 1830,) the progress that dentistry has made in this particular is not difficult to appreciate.

In the successful treatment of teeth when the nerve is exposed much progress has been made; and the efforts have been crowned with such a degree of success that there is reason to hope that the day is not distant when such teeth will be saved and their vitality and life-like appearance preserved.

The variety of instruments used in filling teeth is numberless, so far as excavators and ordinary pluggers are concerned. Automatic mallets, the electric mallet, dental engines with attachments used in the preparation of cavities and finishing fillings have come into use. The rubber dam, given to the profession by Dr. Barnum, as also steel clamps for holding it in place, is one of the most valuable acquisitions that the dentist has received.

It is the opinion of many fully-qualified dentists that there is no necessity for so many dental colleges or even dentists, and that the profession would be elevated and greater progress made if examining boards were established in the several states to prevent the designation "surgeon dentist" being applied, without discrimination, to qualified and unqualified practitioners in this special branch; and this view is strengthened by the fact that not more than 2,500 of the 10,000 now practicing dentistry in the United States are graduates of Dental Colleges. But in the opinion of the writer the number of colleges or of dentists is not to be diminished by ceasing to organize colleges, or graduate dentists, but by establishing other colleges, based on better claims, with more extended and liberal curricula wisely planned and ably carried out, and with preliminary examinations (not as a matter of form, but as a test of general knowledge and mental capacity,) which like Aaron's rod shall swallow up the spurious.

Such colleges will exercise an influence on the dental profession and command the respect of kindred professions; and, in the end, the crude and immature material which fills this new profession will disappear under the teachings and example of eminent practitioners, and a more appreciative judgment of the public. It is only in this way that dentistry can be elevated above a mere mechanical trade. In fact, the profession itself must rise or fall as its practitioners rise or fall in their own scientific attainments. It is very recently that the liberal and educated men of the profession, with a view of making more efficient and useful practitioners who shall have some recommendation in addition to dexterity in the insertion of a filling, have taken an interest in the education of students; dentists, in the greater number of instances, being educated to proficiency in the mechanical rather than the surgical part of the profession. But perfect dentistry requires equal skill and education in both departments. And it is now considered essential that, inasmuch as disease of the teeth is not always a mere local affection, but may, and very generally does, arise from constitutional causes, the average dentist's education should embrace a thorough acquaintance with the anatomical and sympathetic relations of the organs of the mouth with all parts of the system; that he should understand that the welfare of the teeth is ultimately connected with that of the general system, and that he should possess a knowledge of the diseases whose effects may reach these organs. And inasmuch as no two faces are precisely alike, but each has its individual features, the dentist should study the faces of his patients as the artist studies his picture, so that in supplying artificial dentures he may not mar the features he intended to adorn.

Medical men have objected to what they term "fractionally qualified" being made to appear on an equal footing with themselves. A full medical and surgical education was always deemed desirable by those best qualified to judge of it, but the obstacle has been the mechanical acquirement which dentistry required and which would have to be added to a surgeon's qualifications; an arrangement entailing a very protracted period of education. This however, is overcome in these days (at least in Baltimore,) by the dentist pursuing a medical course while in the practice of his profession; and it is likely that ere long practitioners devoting themselves to dental surgery will (like oculists and aurists or obstetricians, or other physicians or surgeons restricting themselves to or selecting one branch of practice in preference to another) be at the same time fully qualified medical men. "The physician, surgeon and dentist have necessarily many practical duties in common; but each has his clearly defined limitation of sphere, requiring specific direction of that general culture which all must possess." And it is now universally accepted that devotion to a specialty of medical art detracts nothing from the position which a man's education and talent entitle him to assume.

As medicine and surgery are combined in the practice of the majority of medical men, so dental surgery and mechanical dentistry are usually practiced together. The day when the dental surgeon and mechanical dentist, like the ophthalmic surgeon and optician, should occupy a separate sphere has not arrived, yet it is an admitted fact that one who works in a laboratory making casts, swaging plates, grinding teeth, soldering them to the plate and finally finishing the whole as neatly as a piece of jewelry work, cannot keep his hands in a condition to perform successfully the delicate manipulative operations required in treating the natural teeth. However, as competition necessitates and stimulates proficiency, more distinctly marked must this division become and the highest excellence in each department be developed.

With the wondrous steps that modern dentistry has taken, the increase in our experience of what can and what cannot be effected by operations, with a full appreciation of the great conservative powers of nature, it must be acknowledged that the faithful student who understands the proper use of medicines and who, being well versed in all the improvements of the day, can use a moderate degree of dexterity in manipulation has before him every incentive for encouragement and success.

The adage "knowledge is power" is emphatically true in the domain of dentistry. He who professes the specialty must possess a knowledge of the improvements, real or fancied, in his science to the very day of his death. He must know that operations which a few years since were deemed totally impracticable can be and are being successfully performed; and he must be able to state frankly and fairly to the patient the contingencies of his case and the result which he may have to expect from a comparison with others of a similar nature.

In thus recounting the many advances made by the dental profession, it may not be out of place to call attention to a few things that have impressed the writer in which there is room for "progress." To begin: the dentist is not just and generous to his brothers in the profession. Dentists are very prone, when called to look over the labors of another, to assume a grave air of shocked superiority and state that "if" such and such a thing had been done, other and better results would have been attained; that a gross violation of all the known principles of surgery has been exhibited and so on. Whereas it is utterly impossible in the majority of cases, after the patient has received treatment, for one unacquainted with the circumstances and contingencies of the case to express any opinion whatsoever concerning it.

Again, there is a tacit acquiescence in all that a patient may say regarding the practice of a brother dentist, which, while it does not commit the listener, yet allows the dissatisfied patient to understand that he is certainly correct in his appreciation of the case. There are those who, as Pope forcibly says:

> "Damn with faint praise, assent with civil leer;
> And without sneering teach the rest to sneer;
> Willing to wound and yet afraid to strike;
> Just hint a fault and hesitate dislike."

This is the most cowardly and miserable kind of defamation. Far better, and far more honest, is the man who boldly speaks his mind than he who, fearing to commit himself, gives the patient further grounds for believing that he has been badly treated and ill-used. Among men of the same profession there is an unavoidable rivalry, so far as they become competitors for the same prize, but in competition there is nothing dishonorable while excellence alone obtains distinction and no advantage is sought by unfair means.

And finally, the laws of barter are applied to brain work and its products. The disastrous influence of this competition in price is fatal to all progress in the art or advancement in the science of dentistry, and is in fact undermining its very foundations. "Dentistry thus learned and thus practiced has no just claim to be called a profession: it has neither the liberality, generosity nor culture which men are accustomed to associate with professional life. The client cannot estimate the cost of his lawyer's pleading, nor can the patient know till long afterwards the full value of his physician's prescription. The conditions of honest barter are absent, for client and patient are alike dependent upon the integrity of the professional man; hence professional bargaining is dishonorable and inevitably leads to the rendering of a disreputable grade of service."

